# Loss of TIPE2 Has Opposing Effects on the Pathogenesis of Autoimmune Diseases

**DOI:** 10.3389/fimmu.2019.02284

**Published:** 2019-09-24

**Authors:** Ruiling Liu, Xiaozhen He, Wenwen Geng, Ting Wang, Qingguo Ruan

**Affiliations:** ^1^Center for Antibody Drug, Institute of Biomedicine and Biotechnology, Shenzhen Institutes of Advanced Technology, Chinese Academy of Sciences, Shenzhen, China; ^2^University of Chinese Academy of Sciences, Beijing, China; ^3^School of Medicine and Life Sciences, University of Jinan, Shandong Academy of Medical Sciences, Jinan, China; ^4^State Key Laboratory Cultivation Base, Shandong Provincial Key Laboratory of Ophthalmology, Shandong Eye Institute, Shandong First Medical University & Shandong Academy of Medical Sciences, Qingdao, China

**Keywords:** TIPE2, psoriasis, IL-17A, migration, EAU

## Abstract

Autoimmune diseases are a physiological state wherein immune responses are directed against and damage the body's own tissues. Cytokines secreted by infiltrated inflammatory cells contribute to the pathogenesis of autoimmune diseases. TIPE2, one of the four family members of Tumor necrosis factor-α induced protein-8 (TNFAIP8), is a negative regulator of innate and adaptive immunity and plays essential roles in the maintenance of immune tolerance. However, studies on the role of TIPE2 during the development of autoimmune diseases have generated contradictory results. In the current study, we sought to determine the role of TIPE2 during the development of IMQ-induced psoriasis and Experimental Autoimmune Uveitis (EAU) in mice. Our study revealed that, while TIPE2-deficiency alleviates psoriasis, it exacerbates the development of EAU. Further studies demonstrated that, although TIPE2-deficient T cells produced more IL-17A, they do not migrate efficiently to the local inflammatory site, i.e., the skin. This in turn led to the decreased IL-17A production in the skin and consequently reduced the severity of psoriasis in TIPE2-deficient mice. However, although TIPE2-deficient T cells still produced more IL-17A in EAU model, they migrate into the inflamed eye as efficient as TIPE2-sufficient T cells, and consequently exacerbates the development of EAU in TIPE2-deficient mice. Taken together, these results indicate that TIPE2 may either promote or suppress autoimmunity depending on the specific inflammatory microenvironment in different types of autoimmune diseases.

## Introduction

Autoimmune disease occurs when healthy body tissues were mistakenly attacked and destroyed by body's own immune system. More than 80 types of different autoimmune diseases have been reported, including psoriasis, Experimental Autoimmune Uveitis (EAU), type 1 diabetes, Experimental Autoimmune Encephalomyelitis (EAE), and Rheumatoid Arthritis (RA). Besides genetic and environmental factors, immunological factor often plays important roles during the pathogenesis of autoimmune disease which triggers the initiation of the disease as well as maintaining the disease state (1, 2). T cells, especially the IL-17A-producing Th17 cells, have been shown to play a dominant role during the pathogenesis of multiple autoimmune diseases including psoriasis and EAU (3–8). Following priming, Th17 cells migrate to local inflammatory site which was mediated by tissue selective integrins and chemokine receptors (9). While IL-17A knockout mice develop significantly less severe psoriasis and EAU (10, 11), treatment with anti-IL-17A antibody markedly reduced inflammation in both psoriatic and EAU mice (12–14).

Tumor necrosis factor-α induced protein-8-like 2 (TNFAIP8L2 or TIPE2) is one member of the TNFAIP8 family. Current study suggests that TIPE2 is a negative regulator of innate and adaptive immune responses (15). TIPE2 is preferentially expressed by immune cells and TIPE2-deficient cells are hyper-responsive to Toll-like receptor (TLR) and T cell receptor (TCR) activation. Further study revealed that TIPE2 may inhibit the activation of NF-κB in T cells and macrophages (15). Since TIPE2 is a negative regulator of immune response, it may play important roles during the pathogenesis of autoimmune disease. In fact, TIPE2 down-regulation was found in the peripheral blood mononuclear cells from patients with Systemic Lupus Erythematosus (SLE) (16). In 2016, Xu et al. reported that TIPE2 alleviates experimental SLE through the induction of M2 macrophage (17). In addition, decreased TIPE2 expression was also found to be correlated with the occurrence and development of RA (18). However, researchers have also found that TIPE2 promotes the development of colitis (19). Recently, Fayngerts et al. reported that TIPE2-deficient leukocytes were defective in polarization and chemotaxis, and consequently TIPE2-deficient mice were resistant to EAE which was mediated by leukocyte (20). Thus, the role of TIPE2 during the development of autoimmune diseases remains elusive.

In the current study, we sought to determine the role of TIPE2 during the development of IMQ-induced psoriasis and EAU in mice. Our study revealed that, although TIPE2 exacerbates the development of psoriasis through promoting the directional migration of T cells to the site of inflammation, it alleviates EAU through the suppression of IL-17A production by T cells.

## Materials and Methods

### Animals

Four to six week-old wild-type and TIPE2-deficient mice in the C57BL/6 background were used in the experiments and kept under pathogen-free conditions at the animal core facility of the Shenzhen Institutes of Advanced Technology, Chinese Academy of Sciences. All efforts were made to minimize the number of mice used and to less animal distress, pain, and injury. Carbon dioxide (CO_2_) was used to euthanize mice. All procedures were preapproved by the Animal Care and Use Committee of Shenzhen Institutes of Advanced Technology, Chinese Academy of Sciences.

### Induction and Clinical Evaluation of Psoriasis

Commercially available IMQ cream (Aldara, Inova pharmaceuticals, USA) was smeared on the shaved back of wild-type and TIPE2-deficient mice in the C57BL/6 background at 4–6 weeks of age as previously reported (21). Alternatively, CD3^+^ T cells were isolated from wild-type and TIPE2-deficient mice and adoptively transferred into *Rag1*^−/−^ mice (10 × 10^6^/mouse). IMQ cream was then used to induce psoriasis as mentioned above. The severity of inflammation of the back skin was evaluated according to the clinical psoriasis area and severity index (PASI) (22, 23). In order to obtain histological profiles of the back skin, skin sections were first fixed in 10% formalin, and then embedded in paraffin, sectioned, and stained with hematoxylin/eosin. Skin sections were finally examined by microscopy.

### Induction and Clinical Evaluation of EAU

EAU was induced by active immunization as previously described (24). Briefly, mice were immunized with IRBP1-20 (4 mg/ml, GPTHLFQPSLVLDMAKVLLD, purchased from China Peptides) emulsified 1:1 in complete Freund's adjuvant (Chondrex, USA) with an additional 100 μl mycobacterium tuberculosis H37R (2.5 mg/ml, BD biosciences, USA) at the base of the tail and 50 μl in each thigh (400 μg peptide per mouse). An additional 200 μg bordetella pertussis toxin (Abcam, USA) was intravenously injected immediately after peptide injection. Eyes were harvested 21 days after immunization and stained with hematoxylin/eosin, and the severity of EAU was evaluated on a scale of 0–4 using previously published criteria (25).

### RNA Isolation and RT-PCR

To prepare total skin cells, the back skin of the mice were collected and washed with cold washing buffer (PBS + 2% FBS), then cut into small pieces and incubated in DMEM supplemented with 0.5 mg/ml collagenase D (Sigma, USA), 0.02 mg/ml DNase (Roche, USA), and 0.1 mg/ml Dispase (Sigma, USA) for 20 min at 37°C with gentle shaking. Supernatant was collected and filtered through a 70-μm strainer (BD Biosciences, USA) to obtain single-cell suspension. Total RNA was isolated using TRIzol reagent following manufacturer's instructions (Life Technologies, USA). RNA samples were reversely transcribed using the primescript reverse transcription kit (Takara, JPN). The expression of chemokines was determined by quantitative RT-PCR using specific primers and Applied Biosystems 7500 system. When determining the relative level of gene expression, GAPDH was used as the internal control. The primers for the detection of mouse chemokines and GAPDH were synthesized as previously described (26, 27).

### ELISA Assay

For cytokine assays, purified CD4^+^ T cells from spleen and total cells from inguinal lymph node (ILN), cervical lymph node (CLN), spleen or eye were cultured at 1 × 10^6^ cells/well in 100 μl of complete DMEM culture medium (Corning, USA) in the presence or absence of anti-CD3 (ebioscience, USA) and/or anti-CD28 (ebioscience, USA). The concentrations of anti-CD3 and anti-CD28 were indicated within the figure or figure legend. Culture supernatants were collected after 48 h; For the preparation tissue extract, total cells from the back skin of the mice were homogenized in 1% chaps (Sigma, USA) supplemented with complete protease inhibitor mixture (Roche, USA). The concentration of IL17A in the cell culture supernatant and tissue extract was determined by quantitative enzyme-linked immunosorbent assay (ELISA) per manufacturer's instructions (eBioscience, USA).

### Antibodies and Flow Cytometry

Flow cytometric analyses were performed on freshly isolated cells from skin, blood, ILN, CLN, and eye. Cells were labeled with a combination of the following fluorescence-conjugated mouse mAbs: PE-anti-CD4, APC-anti-CD8, PerCP-Cy5.5-anti-CD3, APC-anti-CD183(CXCR3), APC-anti-CD184 (CXCR4), PE-anti-CD194 (CCR4), APC-anti-CD195(CCR5), PerCP/Cy5.5-anti-CD196 (CCR6), APC-anti-CD197(CCR7), and FITC-anti-CD199 (CCR9). All antibodies were purchased from BioLegend. For the intracellular staining of IL-17A, cells from the skin or eye were stimulated with PMA + ionomycin (eBioscience, USA) in the presence of GolgoStop (1:1,500 dilution, BD, USA) for 6 h. Cells were then collected and first stained with anti-CD3. After fixation and permeabilization, cells were then stained with anti-IL-17A (BioLegend, USA) per manufacturer's instructions (Invitrogen, USA). For the intracellular staining of phospho-IκBα, cells from the skin or eye were first stained with anti-CD3. After fixation and permeabilization, cells were then stained with purified anti-phospho-IκBα (Ser32/36) antibody (CST, USA), followed by staining with DyLight™ 488-conjugated goat anti-mouse IgG (BioLegend, USA) per manufacturer's instructions (Biolgend, USA). Stained cells were then examined on CytoFLEX flow cytometry system (Beckman Coulter Inc., USA).

### Transwell Migration Assay

Lymphocytes were isolated from blood using mouse peripheral blood lymphocyte isolation kit (Tian Jin Hao Yang Biological Manufacture CO., LTD, China). Total T cells were isolated from blood lymphocyte and ILN using EasySep™ Mouse T Cell Isolation Kit (Stemcell, USA) and “rest” in RPMI1640 culture medium for 1 h. Migration assays were then performed using Transwells. Briefly, cells were placed on the upper layer of a cell permeable membrane of 24-well transwell plate (5 μm pore size, Corning, USA) at 5 × 10^4^/well. IP10 (10 ng/ml, Biolegend, USA), CCL19 (10 ng/ml, Biolegend, USA), or PBS (control group) was placed below the cell permeable membrane. Following an incubation period of 3 h, the cells that have migrated through the membrane are counted by TC10 automated cell counter (Bio-Rad, USA). The chemotaxis index was calculated by dividing the number of cells that migrated in response to chemoattractant with the number of cells that migrated in the control group.

### Confocal Microscopy Analysis

Total T cells were isolated from the ILN using EasySep™ Mouse T Cell Isolation Kit (Stemcell, USA). After resting in RPMI1640 culture medium for 1 h, cells were subjected to point-source stimulation with IP10 (Biologend, USA) at 1 μg/ml for 3 min at 37°C. Cells were then fixed with 4% paraformaldehyde for 15 min at room temperature, permeabilized with 0.1% TritonX-100 in PBS for 10 min and blocked with 5% normal rat serum plus 3% BSA for 1 h at room temperature. After fixation and permeabilization, cells were incubated overnight with a 1:100 dilution of Phospho-AKT (T308) antibody (Cell Signaling Technology, USA) and a 1:1,000 dilution of Phalloidin-iFluor647 (CST, USA). Cells were then washed three times and incubated for 30 min with 1:1,000 diluted DyLight™ 488 Donkey anti-rabbit IgG antibody (Biolegend, USA). Stained cells were then washed, dried and covered with ProLong Gold with DAPI (Invitrogen, USA). Images were acquired using a Leica TCS SP5 confocal microscope and analyzed using ImageJ software.

### Statistical Analysis

The significance of the difference in disease severity was determined by Mann-Whitney *U*-test. The skin thickness, mean florescence intensity (MFI), level of cytokines and percentage of cells were analyzed by Student's *t*-test.

## Results

### TIPE2-Deficiency Alleviates Psoriasis but Promotes the Development of EAU

**Figure 1:**
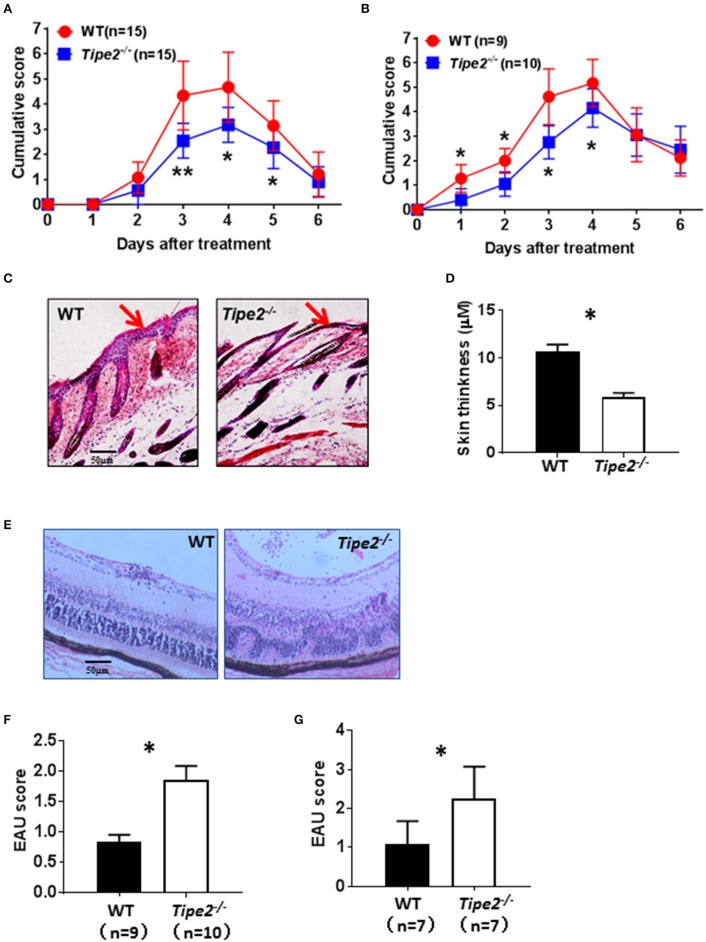
TIPE2-deficiency alleviates psoriasis but promotes the development of EAU. **(A)** WT (*n* = 15) and TIPE2-deficient (*n* = 15) mice in the C57BL/6 background were treated with IMQ cream on the shaved back skin as mentioned in the Materials and Methods. Mice were monitored for the development of erythema and scaling on the back skin. The cumulative score (erythema plus scaling) is depicted. Data were combined from two separate experiments. **(B)** Total T cells were isolated from wild-type and TIPE2-deficient mice and adoptively transferred to *Rag1*^−/−^ mice (10 × 10^6^/mouse). IMQ cream was used to induce psoriasis as mentioned in the Materials and Methods. The cumulative score (erythema plus scaling) was calculated as mentioned in **(A)**. Data were combined from two separate experiments. **(C)** Mice from **(A)** were killed 6 days after the first IMQ treatment and H&E staining of the back skin of mice was performed. **(D)** Skin thickness was measured after H&E staining. **(E)** EAU was induced in WT (*n* = 9) and TIPE2-deficient (*n* = 10) mice as described in the Materials and Methods. Twenty-one days after immunization, H&E staining of the eye section was performed. Data were combined from two separate experiments. **(F)** Mice were treated as in **(E)** and EAU scores were determined on a scale of 0–4 based on the number, type, and size of lesions. **(G)** Total T cells were isolated from wild-type and TIPE2-deficient mice and adoptively transferred to *Rag1*^−/−^ mice (10 × 10^6^/mouse). EAU was induced as described in the Materials and Methods and characterized as in **(F)**. ^*^P < 0.05; ^**^P < 0.01.

IMQ is a ligand for TLR7/8 that when applied topically to the skin, induces psoriasis-like inflammation in susceptible mice including BABL/c and C57BL/6. The IMQ-psoriasis closely resembles human psoriasis lesions with respect to phenotypic and histological characteristics. EAU is the animal model of human uveitis that most closely resembles sympathetic ophthalmia. It is the most widely used and well-studied uveitis model. In order to determine the roles of TIPE2 in the pathogenesis of different types of autoimmune diseases, we established IMQ-induced psoriasis model and EAU model in wild-type and TIPE2-deficient mice in the C57BL/6 background. As shown in Figure 1A, IMQ-induced skin inflammation in TIPE2-deficient mice resulted in lower scores for erythema and scaling at the peak-stage of the disease. The difference is small but significant. Although IMQ-induced psoriasis is a T cell–dependent disease, other hematopoietic cell types may also contribute to the pathogenesis. In order to directly test the T cell–specific function of TIPE2 in psoriasis, we studied the IMQ-induced psoriasis in *Rag1*^−/−^ mice that had received WT or TIPE2-deficient T cells. We found that mice received TIPE2-deficient T cells developed less severe symptoms than those reconstituted with WT T cells (Figure 1B). Consistent with these clinical findings, H&E-stained sections of skin from TIPE2-deficient mice demonstrated decreased epidermal thickening by Figures 1C,D. In contrast to the phenotype difference displayed in IMQ-induced psoriasis model, TIPE2-deficient mice exhibited more severe retinal edema and structural distortion in the EAU model (Figure 1E). When pathological changes in the retina were evaluated according to the scoring criteria previously reported, WT mice had a mean score of 0.8, whereas that number for TIPE2-deficient mice is 1.8 (Figure 1F). Similar phenotypical difference was also detected when EAU was induced in mice adoptively transferred with T cells from either WT or TIPE2-deficient mice (Figure 1G). Since both IMQ-induced psoriasis and EAU are T-cell mediated autoimmune disease animal model and our study has also established that TIPE2 in T cells regulates the pathogenesis of both psoriasis and EAU, in this study we focused on the roles of TIPE2 in T cells in regular IMQ-induced psoriasis model and EAU model.

### TIPE2 Suppresses the Production of IL-17A by T Cells

**Figure 2:**
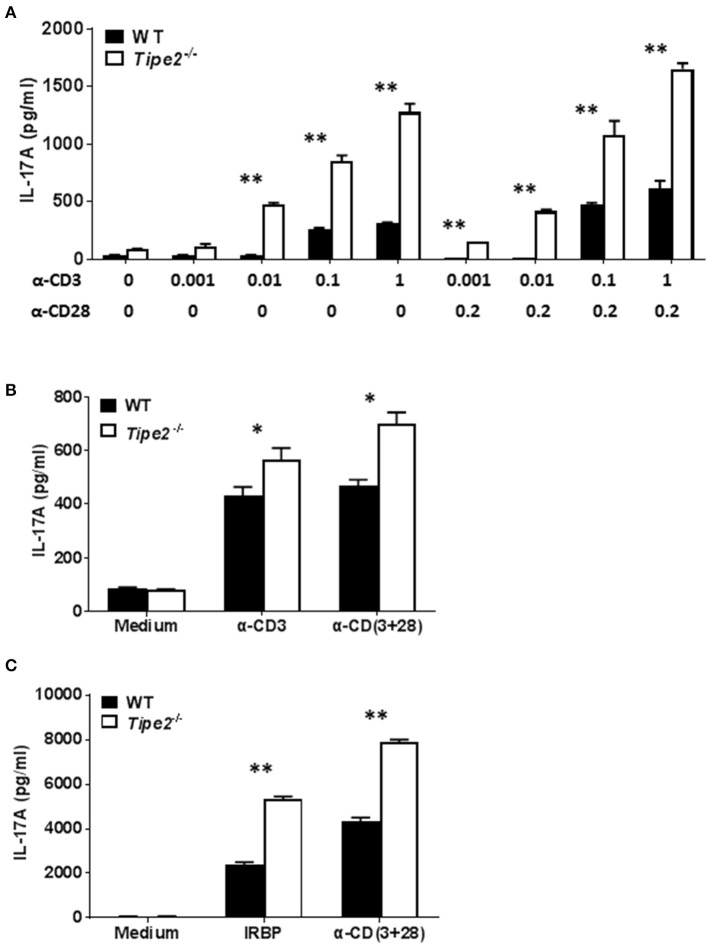
TIPE2 suppresses IL-17A production by splenic T cells. **(A)** CD4^+^ T cells were isolated from untreated WT (*n* = 3) and TIPE2-deficient mice (*n* = 3) and cultured with or without different concentrations (μg/ml) of plate-bound anti-CD3 plus soluble anti-CD28 as indicated. After 48 h, culture supernatants were collected and the concentration of IL-17A was determined by ELISA. Data are representative of three separate experiments. **(B)** WT (*n* = 5) and TIPE2-deficient (*n* = 5) mice were treated as in Figure 1A and killed 3 days after the first IMQ treatment. Total splenocytes were isolated and cultured at 1 million per well in 96-well plate with or without plate-bound anti-CD3 (0.5 μg/ml) or/and anti-CD28 (0.5 μg/ml). After 48 h, the concentration of IL-17A in the culture supernatants was determined by ELISA. Data are representative of two separate experiments. **(C)** EAU was induced in WT (*n* = 4) and TIPE2-deficient (*n* = 5) mice as in Figure 1E and killed 21 days following immunization. Total splenocytes were isolated and cultured at 1 million per well in 96-well plate with or without IRBP (30 μg/ml) or anti-CD3 (0.5 μg/ml) plus anti-CD28 mAb (0.5 μg/ml). After 48 h, the concentration of IL-17A in the culture supernatants was determined by ELISA. Data are representative of two separate experiments. ^*^P < 0.05; ^**^P < 0.01.

Both IMQ-induced psoriasis and EAU model contains a strong T cell component and the disease development is dependent on IL-17A. To determine the potential effect of TIPE2 deficiency on the production of IL-17A by T cells, we examined the expression of IL-17A produced by T cells *in vitro*. First we isolated CD4^+^ T cells from the spleen of untreated WT and TIPE2-deficient mice and cultured them *in vitro* with or without different concentrations of plate-bound anti-CD3 and soluble anti-CD28. We found that TIPE2 deficient T cells produced significantly higher level of IL-17A (Figure 2A). Secondly, we isolated total splenocyte from IMQ-treated WT and TIPE2-deficient mice and cultured them *in vitro* with or without plate-bound anti-CD3 and/or soluble anti-CD28. We found that the expression of IL-17A was also significantly increased by TIPE2-deficient T cells (Figure 2B). Thirdly, we isolated total splenocyte from WT and TIPE2-deficient mice that have been treated to induce EAU. Cells were then cultured *in vitro* with or without IRBP or anti-CD3 plus anti-CD28. Again we found that TIPE2-deficient T cells produced significantly more IL-17A (Figure 2C). Taking together, these results indicate that TIPE2 is a negative regulator of IL-17A expression in T cells.

### The Production of IL-17A Is Decreased in the Skin but Increased in the Eye of TIPE2-Deficient Mice

**Figure 3:**
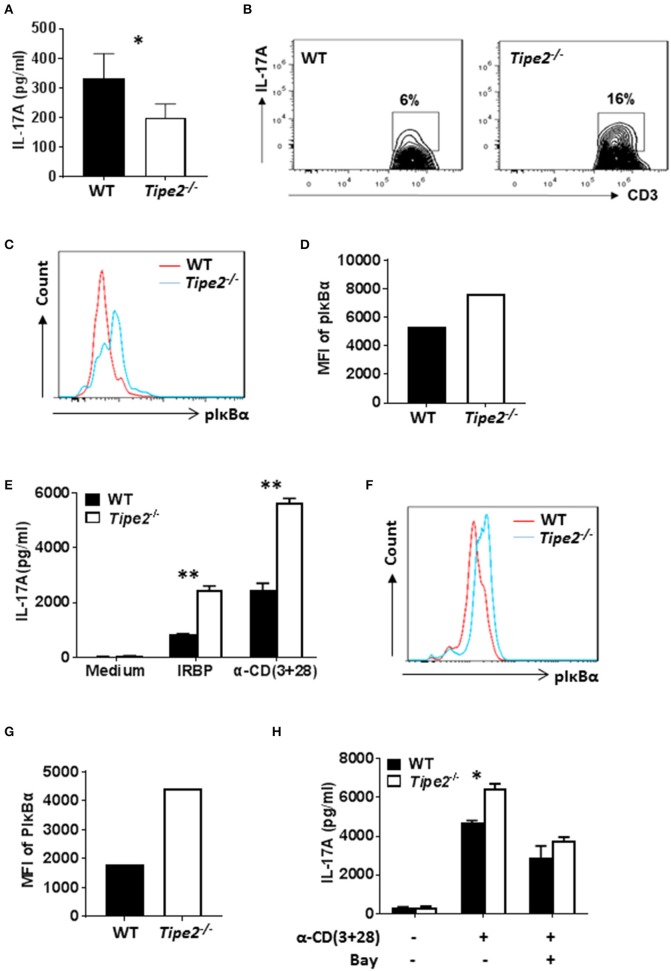
The production of IL-17A is decreased in the skin but increased in the eye of TIPE2-deficient mice. **(A–D)** WT (*n* = 5) and TIPE2-deficient (*n* = 5) mice were treated as in Figure 1A and killed 3 days after the first IMQ treatment. **(A)** Tissue extracts were prepared from the back skin and the concentration of IL-17A in the tissue extract was determined by ELISA. **(B)** Total cells from the back skin were isolated and stimulated *in vitro* with PMA+ionomycin in the presence of GolgiStop for 6 h. Cells were first stained with anti-CD3, then fixed, permeabilized and stained with anti-IL-17A and examined by flow cytometry. Cells shown were gated on CD3^+^ T cells. **(C)** Total cells from the back skin were isolated and stained with anti-CD3. After fixation and permeabilization, cells were then stained with anti-phospho-IκBα antibody, followed by staining with DyLight™ 488-conjugated goat anti-mouse IgG. Stained cells were then examined by flow cytometry. Cells shown were gated on CD3^+^ T cells. **(D)** Relative expression level of phosphorylated IκBα was shown as the mean fluorescence intensity (MFI). **(E–H)** WT (*n* = 4) and TIPE2-deficient (*n* = 5) mice were treated as in Figure 1E. Mice were killed 21 days after the immunization and total cells were isolated from the eye. **(E)** Cells were cultured with or without IRBP (30 μg/ml) or anti-CD3 (0.5 μg/ml) plus anti-CD28 mAb (0.5 μg/ml). After 48 h, the concentration of IL-17A in the culture supernatants was determined by ELISA. **(F)** The level of phosphorylated IκBα was determined as in **(C)**. **(G)** Relative expression level of phosphorylated IκBα was shown as the mean fluorescence intensity (MFI). **(H)** Cells were cultured with or without plate-bound anti-CD3 (0.5 μg/ml) plus soluble anti-CD28 (0.5 μg/ml). In addition, NF-κB inhibitor BAY11-7082 (1 μM) was used to inhibit NF-κB activity as indicated. After 48 h, level of IL-17A in the supernatants was determined by ELISA. Data are representative of two separate experiments. ^*^P < 0.05; ^**^P < 0.01.

To investigate whether TIPE2 deficiency alters IL-17A production in the inflamed skin, tissue extracts were prepared from the skin of IMQ-treated WT and TIPE2-deficient mice and the production of IL-17A was measured by ELISA. We found that the production of IL-17A was significantly decreased in the skin from TIPE2-deficient mice (Figure 3A). This result is in consistency with the phenotype that TIPE2-deficiency alleviates IMQ-induced psoriasis. However, when flow cytometry was used to examine the percentage of IL-17-producing cells within gated CD3^+^ T cells, we found that the percentage of IL-17A-producing cells was much higher in TIPE2-deficient mice (Figure 3B). In addition, we also found that the phosphorylation of Iκbα was significantly increased in TIPE2-deficient T cells (Figures 3C,D). Similar experiments were performed with the cells isolated from the inflamed eye of WT and TIPE2-deficient mice that have been treated to induce EAU. In consistency with the phenotype that TIPE2-deficiency promotes the development of EAU, the production of IL-17A (Figure 3E) and the phosphorylation of Iκbα (Figures 3F,G) was significantly increased by TIPE2-deficient T cells. In addition, we also found that WT and TIPE2-deficient T cells no longer displayed difference in IL-17A expression after treatment with NF-κB inhibitor BAY11-7082 (Figure 3H). Taking together, these results indicate that TIPE2 may suppress IL-17A production by T cells through the inhibition of NF-κB activation.

### The Percentage of T Cells in TIPE2-Deficient Mice Is Decreased in the Skin but Comparable in the Eye Compared With That in WT Mice

**Figure 4:**
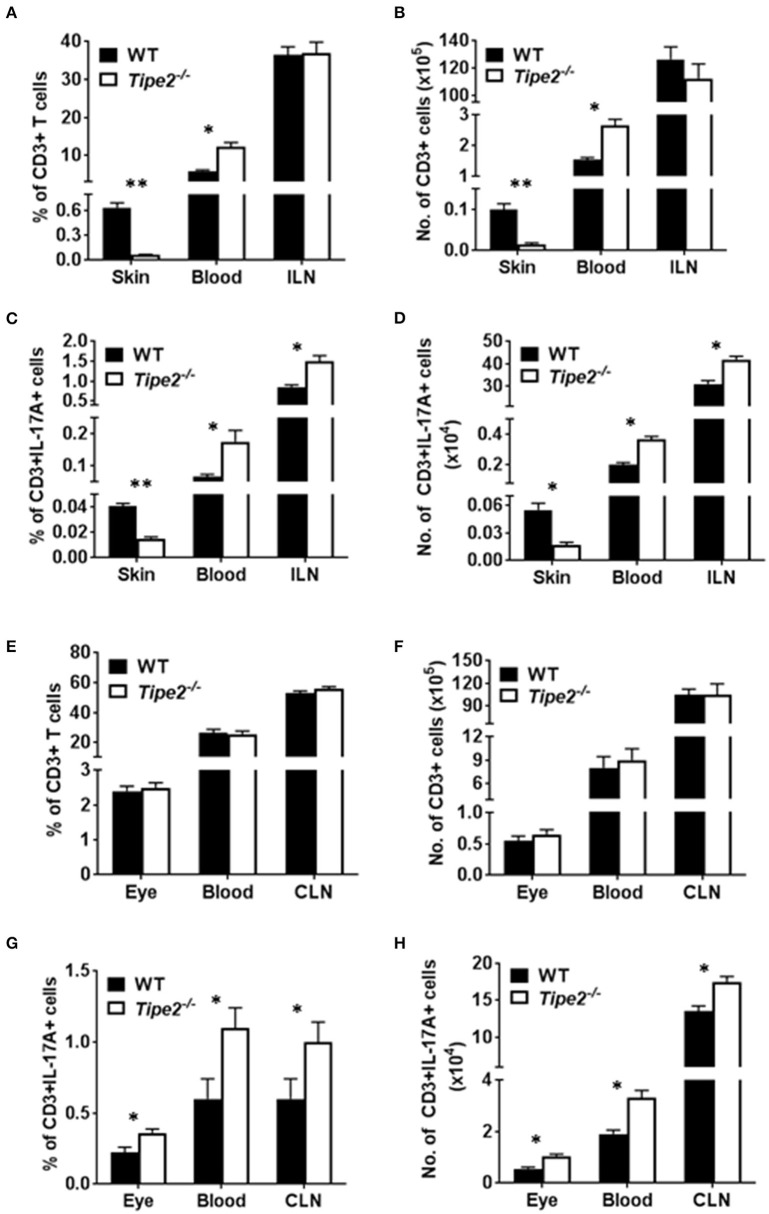
Comparison of the percentage and absolute number of CD3^+^ and CD3^+^IL-17A^+^ T cells in WT and TIPE2-deficient mice. **(A–D)** WT (*n* = 5) and TIPE2-deficient (*n* = 5) mice were treated as in Figure 1A and killed 3 days after the first IMQ treatment. The percentage **(A)** and absolute number **(B)** of CD3^+^ T cells in the skin (0.8 cm^2^), blood (0.6 ml) and ILN were determined by cell surface staining with fluorescent labeled antibody against CD3. The percentage **(C)** and number **(D)** of CD3^+^IL-17A^+^ T cells in the skin (0.8 cm^2^), blood (0.6 ml) and ILN was determined by cell surface staining as described above followed by intracellular staining with fluorescent labeled antibody against IL-17A. **(E–H)** WT (*n* = 6) and TIPE2-deficient (*n* = 7) mice were treated as in Figure 1E. Mice were killed 21 days after the immunization and the percentage and number of CD3^+^ **(E,F)** and CD3^+^IL-17A^+^ T cells **(G,H)** in the eye (one eye), blood (0.6 ml) and CLN were determined as described above. ILN, inguinal lymph node; CLN, cervical lymph node. ^*^P < 0.05; ^**^P < 0.01.

Although TIPE2-deficient T cells produced more IL-17A, TIPE2-deficient mice produced much less IL-17A in the skin and displayed reduced severity of IMQ-induced psoriasis. To solve this paradox, we examined the percentage and absolute number of T cells in IMQ-treated WT and TIPE2-deficient mice. The results showed that, while the percentage (Figure 4A) and absolute number (Figure 4B) of CD3^+^ T cells were significantly increased in the blood from TIPE2-deficient mice, they were significantly decreased in the skin. The percentage and absolute number of CD3^+^ T cells were comparable in the draining lymph node (inguinal lymph node, ILN) between WT and TIPE2-deficient mice (Figures 4A,B). Because we have shown that TIPE2 suppresses IL-17A production by T cells during the development of IMQ-induced psoriasis (Figure 2B), the percentage (Figure 4C) and absolute number (Figure 4D) of CD3^+^IL-17A^+^ T cells was significantly increased in the blood and ILN as expected. However, the percentage and absolute number of CD3^+^IL-17A^+^ T cells was significantly decreased in the skin (Figures 4C,D). We think that this is due to the decreased percentage and number of CD3^+^ T cells found in the skin from TIPE2-deficient mice, despite the fact that the proportion of IL-17-producing cells within gated CD3^+^ T cells was much higher in the skin from TIPE2-deficient mice (Figure 3B). This may also explain why the net production of IL-17A was significantly decreased in the skin from TIPE2-deficient mice (Figure 3A). As for the EAU model, no significant difference was found in the inflamed eye as well as in the blood and draining lymph node (cervical lymph node, CLN) between WT and TIPE2-deficient mice in the percentage and absolute number of CD3^+^ T cells (Figures 4E,F). However, because we have shown that TIPE2 suppresses IL-17A production by T cells during the development of EAU (Figure 2C), the percentage and absolute number of CD3^+^IL-17A^+^ T cells was significantly increased in the eye as well as in the blood and CLN from TIPE2-deficient mice (Figures 4G,H). Taken together, these results indicate that, although TIPE2-deficient T cells produced more IL-17A in both disease models, they are defective in migration to the skin in IMQ-induced psoriasis model but not to the eye in EAU model. Next we focused on the roles of TIPE2 in regulating T cell migration during the development of IMQ-induced psoriasis.

### T Cells From IMQ-Treated TIPE2-Deficient Mice Express Normal Levels of Chemokine Receptors but Are Defective in Chemotaxis *in vitro*

**Figure 5:**
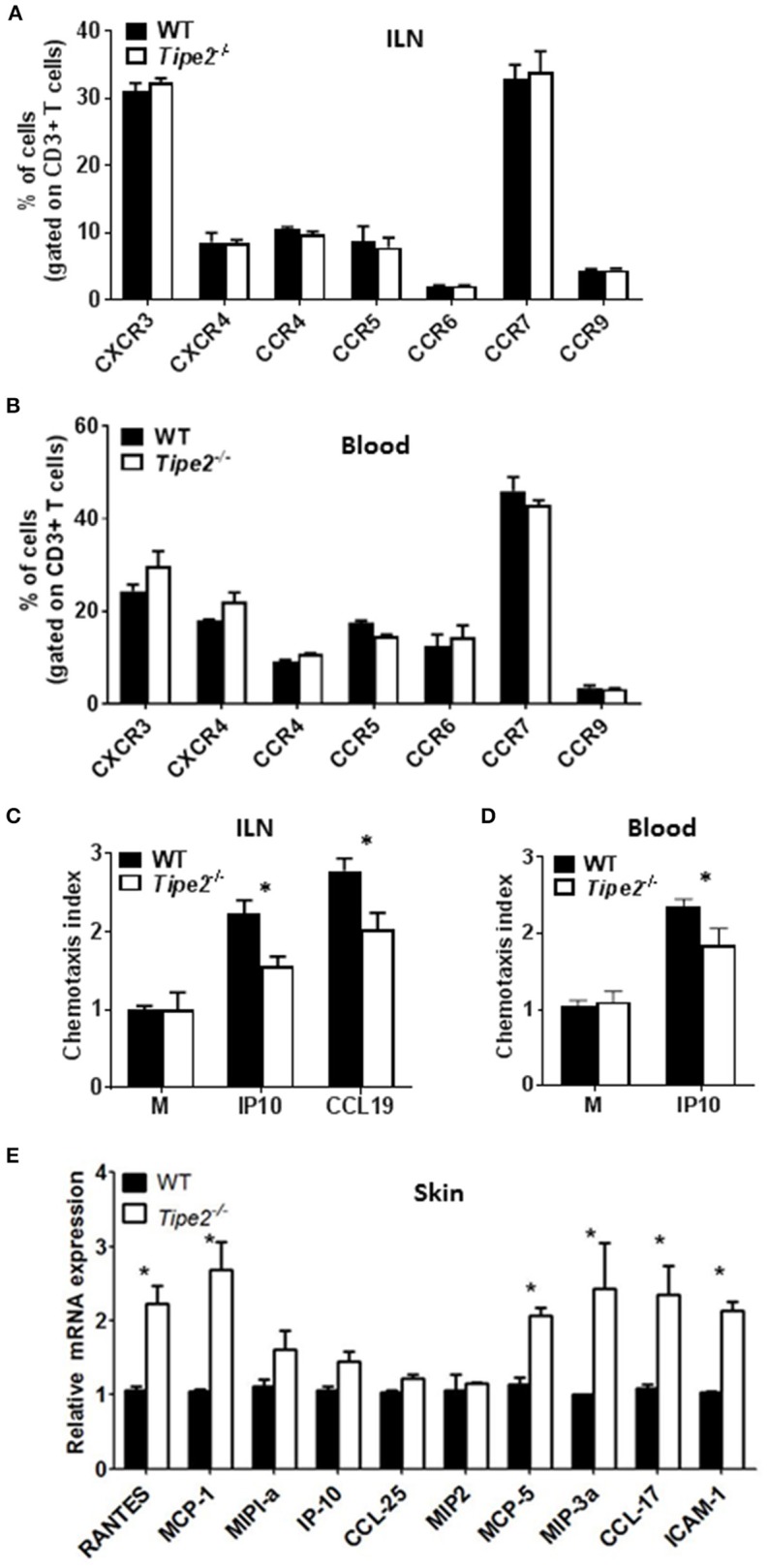
T cells from TIPE2-deficient mice express normal levels of chemokine receptors but are defective in chemotaxis *in vitro*. WT (*n* = 5) and TIPE2-deficient (*n* = 5) mice were treated as in Figure 1A and killed 3 days after the first IMQ treatment. **(A)** Total cells were isolated from ILN and the expression of indicated chemokine receptors on the surface of CD3^+^ T cells was determined by cell surface staining with florescence-labeled antibodies against CD3 and chemokine receptors. Data shown are for gated CD3^+^ T cells. **(B)** Lymphocytes were isolated from blood and the expression of indicated chemokine receptors on the surface of CD3^+^ T cells was determined as in **(A)**. Data shown are for gated CD3^+^ T cells. **(C,D)** Total T cells were isolated from ILN **(C)** and blood lymphocyte **(D)**. *in vitro* transwell assay was performed to determine the migratory capacity of T cells toward IP10 and CCL19, which are the ligand for CXCR3 and CCR7, respectively. The chemotaxis index was calculated as mentioned in the Materials and Methods. **(E)** Total RNA was extracted from the back skin, and the mRNA expression of indicated chemokines was determined by quantitative RT-PCR. Data are representative of two separate experiments. ILN: inguinal lymph node. ^*^P < 0.05.

During the development of psoriasis, mature activated T cells migrate through the postcapillary venules into the dermis after establishing molecular interactions mediated by tissue selective integrins and chemokine receptors (homing receptors). To determine how TIPE2 affects T cell migration during the development of psoriasis, we examined the expression of multiple chemokine receptors on the surface of CD3^+^ T cells from the ILN and blood of IMQ-treated WT and TIPE2-deficient mice. Our results showed that the there is no significant difference in the expression of CXCR3, CXCR4, CCR4, CCR5, CCR6, CCR7, and CCR9 between IMQ-treated WT and TIPE2-deficient mice (Figures 5A,B). However, *in vitro* chemotaxis assay showed that TIPE2-deficient T cells from ILN were defective in migration toward IP10 and CCL19 (Figure 5C), which are the ligand for CCR7 and CXCR3, respectively. Similar results were found with the cells from the blood of IMQ-treated mice (Figure 5D). In order to exclude the possibility that decreased local chemokine production led to the defective migration of TIPE2-deficient T cells to the skin, we also examined the expression of multiple chemokines in the skin. Our results revealed that the mRNA expression of most of the chemokines was not decreased but even increased in the skin from TIPE2-deficient mice (Figure 5E). Because most of those chemokines were direct targets of NF-κB, it is possible that TIPE2 suppresses the production of chemokine in the skin through the inhibition of NF-κB activity.

### T Cells From IMQ-Treated TIPE2-Deficient Mice Are Defective in Leading-Edge Formation During Chemotaxis

**Figure 6:**
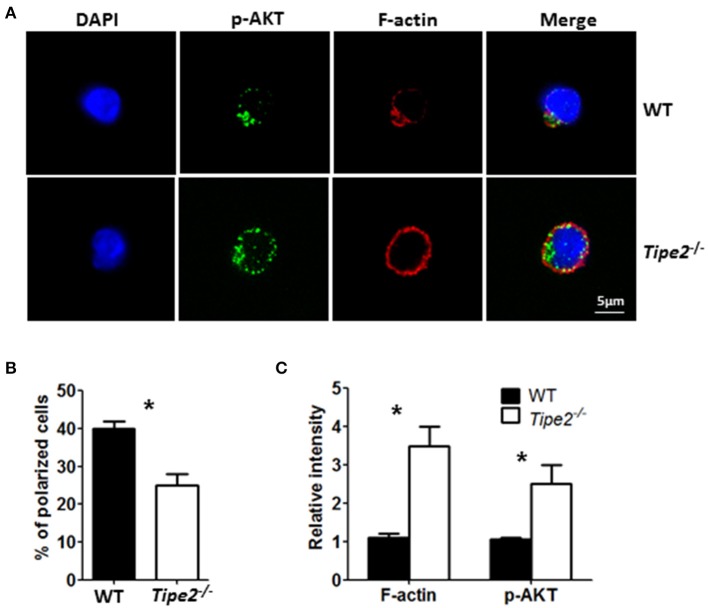
TIPE2-deficient T cells are defective in leading-edge formation during chemotaxis. WT (*n* = 5) and TIPE2-deficient (*n* = 5) mice were treated as in Figure 1A and killed 3 days after the first IMQ treatment. Total T cells were isolated from inguinal lymph node and then subjected to point-source stimulation with IP10 at 1 μg/ml for 3 min at 37°C and stained with Phalloidin, anti-phospho-AKT antibody and DNA-binding dye DAPI. **(A)** The distributions of F-actin, phospho-AKT and DAPI in WT and TIPE2-deficient T cells were assessed by confocal microscopy. Data are representative of two separate experiments. **(B)** The percentages of polarized WT and TIPE2-deficient T cells were calculated. **(C)** The total intensity of F-actin and p-AKT was calculated using ImageJ. For **(B,C)**, data are pooled results from two separate experiments with more than 50 cells analyzed per genotype. ^*^P < 0.05.

Previous studies have shown that TIPE2 promotes leading-edge formation in neutrophils through enhancing phosphoinositide-dependent signaling and cytoskeleton remodeling. Therefore, we examined the polarization of F-action and phosphor-AKT (T308) in CD3^+^ T cells isolated from the ILN of IMQ-treated WT and TIPE2-deficient mice in response to point-source stimulation with IP10. Our results demonstrated that, although the distribution of F-actin and phosphor-AKT was aggregated on the surface of both WT and TIPE2-deficient T cells, more TIPE2-deficient T cells were not polarized (Figures 6A,B). Interestingly, the total amount of F-actin and phosphor-AKT were increased in TIPE2-deficient T cells (Figure 6C). Taking together, these results indicate that, although TIPE2 suppresses the expression of F-actin and phosphor-AKT, it promotes the leading-edge formation during chemotaxis and consequently enhances T cell directional migration.

## Discussion

Although it has been reported that TIPE2 is a negative immune regulator, the exact role of TIPE2 during the development of autoimmune disease remains elusive. Contradictory results have been reported when investigating the role of TIPE2 during the development of different types of autoimmune diseases. Using IMQ-induced psoriasis model and EAU model, our current study revealed that TIPE2 may either promote or suppress autoimmunity, depending on the specific inflammatory microenvironment in different types of autoimmune diseases.

Since TIPE2 functions as a negative regulator of adaptive immune response, we speculate that TIPE2-deficient mice will be more susceptible to developing psoriasis and EAU. However, our current study showed that, while TIPE2-deficient mice develop more severe EAU, they unexpectedly develop less severe IMQ-induced psoriasis. In 2017, Fayngerts et al. reported that TIPE2 functioned as a local enhancer of cytoskeleton remodeling and promoted leading-edge formation in neutrophils (20). Our previous work also showed that TIPE2 enhances the cytoskeleton remodeling and promotes the thymus egress of tTregs (28). In the current study we further confirmed that TIPE2 may promote T cell migration via the same mechanism in the psoriasis model. This would explain why TIPE2-deficient mice develop less severe IMQ-induced psoriasis. We speculate that TIPE2 may play both anti-inflammatory and pro-inflammatory roles during the development of autoimmune diseases. Although T cells produced more IL-17A in both psoriasis and EAU model, they were defective in migration to the skin but not to the inflamed eye. The final outcome depends on the balance between these two opposing effects. This theory may also explain why the IMQ-induced skin inflammation of TIPE2-deficient mice is only marginally reduced compared with that of WT mice.

However, it still remains a question why TIPE2-deficient T cells were defective in migration to the site of inflammation in one particular autoimmune disease but not in another? After priming in the draining lymph node, mature activated Ag-specific T cells migrate through the postcapillary venules into local inflammatory site through a chemotaxis-dependent manner (29). Alternatively, primed T cells may also traffick to local inflammatory site through random migration. For example, random migration contributes to cytotoxicity of activated CD8^+^ T-cells and may be used as a biomarker to predict the effect of immunotherapy using activated lymphocytes (30). Thus, depending on the specific inflammatory microenviroment in different types of autoimmune disease, TIPE2-deficiency may or may not affect the accumulation of T cells in the local inflammatory site. In EAU model, because pro-inflammatory leads to the breakdown of RBB (Retinal-Blood-Barrier), the majority of T cells that accumulated in the flamed eye may migrate in a chemotaxis-independent manner. The total amount of F-actin was increased in TIPE2-deficient T cells further indicates that TIPE2 in T cells may suppress non-directional cell movement.

While EAU in animals was used as a model of human uveitis, treating mice with innate TLR7/8 ligand IMQ rapidly induces dermatitis closely resembling human psoriasis. They are both T-cell mediated autoimmune diseases animal model, because only T cells can adoptively transfer those diseases (11, 14). Studies in these two disease models demonstrated that Th17 rather than Th1 cells play a critical role during their pathogenesis (10, 11). Because IL-17A is the primary Th17 cell effector cytokine, disease development was almost completely blocked in mice deficient for IL-17A or IL-17 receptor (31, 32). *In vivo* neutralization of IL-17A is able to ameliorate EAU and IMQ-induced psoriasis (12–14). While nuclear hormone receptor RORγT is a master regulator of the differentiation of IL-17-producing T cells (33), other factors such as STAT3 and NF-κB have also been shown to regulate the expression of IL-17A (34, 35). TIPE2 negatively regulates immune response through inhibiting the activity of NF-κB in T cells (36). Our results also showed that the level of phosphorylated IκBα was markedly up-regulated in TIPE2-deficient T cells derived from the inflamed eye and skin. Furthermore, we showed that WT and TIPE2-deficient T cells no longer displayed difference in IL-17A expression after treatment with NF-κB inhibitor. These results suggest that TIPE2 may inhibit the expression of IL-17A in T cells by suppressing NF-κB activity.

In addition to Th17 cells, IL-17A is also produced by a variety of cell types from the innate and adaptive immune systems (37, 38). Other types of T cells such as CD8 T cells, NKT cells and Gamma delta (γδ) T cells are also significant potential sources of IL-17A (38–40). In our current study, we didn't distinguish the T cell types in which TIPE2 inhibits the expression of IL-17A. While one group reported that Th17 cells are the major source of IL-17A in psoriatic dermis (41), others showed that the majority of IL-17A in the skin was produced by IL-23-responsive dermal γδ T cells (40, 42). Thus, future study is needed to determine the role of TIPE2 on IL-17A production in different IL-17A-producing T cells.

In summary, our study has revealed that TIPE2 can function as both a negative and positive regulator of autoimmunity. On one hand, TIPE2 could suppress inflammatory response through the inhibition of IL-17A production by T cells. On the other hand, TIPE2 could promote inflammatory response through enhancing the directional migration of T cells. The final outcome may depend on the balance of TIPE2 mediated anti-inflammatory and pro-inflammatory effects.

## Data Availability Statement

All datasets generated for this study are included in the manuscript/supplementary files.

## Ethics Statement

This study was carried out in accordance with the recommendations of the Animal Care and Use Committee of Shenzhen Institutes of Advanced Technology, Chinese Academy of Sciences. The protocol was approved by Shenzhen Institutes of Advanced Technology, Chinese Academy of Sciences.

## Author Contributions

QR and TW designed research and analyzed data. RL, XH, and WG performed research. QR wrote the manuscript.

### Conflict of Interest

The authors declare that the research was conducted in the absence of any commercial or financial relationships that could be construed as a potential conflict of interest.
